# Treatment of acute, severe epigastric/chest pain in a patient with stomach cancer following gastrectomy: A case report

**DOI:** 10.3892/ol.2015.2886

**Published:** 2015-01-19

**Authors:** IWONA ZAPOROWSKA-STACHOWIAK, LIDIA GORZELIŃSKA, MACIEJ SOPATA, JACEK ŁUCZAK

**Affiliations:** 1 Palliative Medicine In-patient Unit, University Hospital of Lord’s Transfiguration, Poznan 60-806, Poland; 2 Department of Pharmacology, Poznan University of Medical Sciences, Poznan 60-806, Poland; 3 Chair and Department of Palliative Medicine, Poznan University of Medical Sciences, Poznan 60-806, Poland

**Keywords:** chest pain, epigastric pain, palliative care, stomach cancer

## Abstract

The treatment of acute chest pain can be a challenge in palliative care. Firstly, because acute chest pain is a symptom of a paucity of diseases, which makes diagnosis difficult and time consuming, while there is also a time constraint, due to the extreme suffering of the patient. Secondly, the condition of a patient with advanced cancer disease and co-morbidities does not always allow for required diagnostic procedures. The present report describes a case of acute, severe epigastric/chest pain in a patient with dynamic disease progression, who was receiving palliative care. This study also demonstrates that the pathophysiology of pain in a terminal patient may determine the treatment strategy. The patient in the present case was a 41-year-old male, who had previously undergone gastrectomy for stomach cancer, followed by postoperative chemotherapy. The patient was treated with palliative chemotherapy for metastases to the lungs, liver and lymph nodes, which led to the development of iatrogenic peripheral neuropathy. The patient was subsequently admitted to the Palliative Medicine In-patient Unit of the University Hospital of Lord’s Transfiguration (Poznan, Poland) with the complaint of acute epigastric and chest pain. An electrocardiogram, echocardiogram, chest and abdomen computerized tomography scan, esophagoduodenoscopy and laboratory analyses were performed to determine the source of the pain. The patient was treated with morphine sulfate, metoclopramide, midazolam, diazepam, acetaminophen, ketamine, hyoscine butylbromide, propofol, dexamethasone and amoxycillin, and received parenteral nutrition. As the source of pain remained unclear, a second esophagoduodenoscopy was performed to determine a diagnosis, resulting in pain relief. Thus, in the present case, esophagoduodenoscopy was diagnostic and therapeutic. Furthermore, although the treatment of acute chest pain may be a challenge in palliative care, the present study indicates that pain treatment should be adjusted to anatomical, pathophysiological and pharmacological factors, and may pose risks due to the unavoidable parenteral co-administration of multiple agents with strong therapeutic effects.

## Introduction

Scenarios designed in accordance with the World Health Organization analgesic ladder are frequently used in palliative medicine (1). Although preliminary diagnoses are based on clinical signs, the pathophysiology of pain is not always sufficiently examined in palliative patients, thus, the diagnosis of acute chest or epigastric pain may be a challenge. Furthermore, the diagnosis and how a patient perceives their symptoms determine the efficacy of the treatment (2). Acute chest pain may be of cardiac origin, the most life-threatening type, or non-cardiac origin, which includes gastrointestinal (GI) and non-GI causes, for example bone, articular, muscular or pulmonary conditions, herpetic, esophageal or psychiatric sources. Non-cardiac acute chest pain may also be caused by drugs including local anaesthetic (for example cocaine or thyroid hormone preparation, such as levothyroxine), anticancer drugs (for example doxorubicine, 5-fluorouracil, trastazumab and paclitaxel), non-steroidal anti-inflammatory drugs (for example naproxen and celecoxib) and antimigraine preparations (for example sumatriptan) (3).

Sixty percent of cases of non-cardiac chest pain are of esophageal origin, resulting from three predominant causes: Gastroesophageal reflux disease, esophageal spasms caused by a disturbed progression of the peristaltic wave or esophageal hypersensitivity (abnormal sensory function) (4). In addition, retrosternal, chest or epigastric pain may stem from inflammation involving the esophagus (5,6) or a dysfunction of visceral sensory neurons (7).

Proton pump inhibitors (PPIs) are used in the treatment of gastroesophageal reflux disease. However, partial responders to PPIs and patients with the other two abovementioned causes of esophageal-induced chest/epigastric pain are treated with pain modulators, including low-dose antidepressants such as, imipramine, sertraline, trazodone, citalopram (5,8–10), theophylline (11,12), nifedipine, diltiazem (5,8,9,13), nitroglycerin, isosorbide nitrate, amyl nitrite (14) and botulinum toxin type A (5,8,9).

The vagus nerve and sympathetic trunk are sources of esophageal plexus (5), and gastrectomy (GE) is associated with the risk of non-selective vagotomy (VT). Vagotomy may result in disturbed, receptive relaxation reflexes and accommodation, causing hypertonic food to rapidly reach the intestine, possibly triggering distension of the intestinal wall (via increased water secretion) and resulting in enzyme and bile salt dilution (15,16). This dilution may generate a defect in heme-iron liberation, impairment of vitamin A, D, E and K absorption, diarrhea and hypovolemia. In addition, this reflex may act via hormones and neurotransmitters to induce nausea or regurgitation, cramps, pain and vasomotor reactions involving tachycardia, palpitations, dysfunctional orthostatic regulation and cutaneous vascular dilation (17). These symptoms, observed 30–60 min following food consumption, are termed early gastric emptying (18,19).

Furthermore, hypertonic food in the intestine contains large quantities of carbohydrates, which are absorbed quickly, causing a fast and high hyperglycemic peak followed by reactive hypoglycemia, which may manifest as confusion, anxiety, nervousness and activation of the sympathetic nervous system, for example vasomotor reactions may cause flushing and tachycardia (20). These symptoms, observed 90–180 min following food consumption, are termed late gastric emptying (19).

Upon swallowing, the vagovagal reflex causes the lower esophageal sphincter to open, however, in case of GI passage disturbances (i.e., due to the presence of a tumor), the peristaltic waves of the esophagus and the sphincter function are disturbed. Food, fluid and mucous are accumulated in the dilated esophagus (undigested food trapping), distending and irritating the esophagus, and causing partially opioid-resistant spasmodic pain. Thus, the process of emptying the esophagus is impaired and regurgitation of food occurs, accompanied by retrosternal pain and salivation. Furthermore, a concurrent dysfunction of the lower esophageal sphincter results in no protection against gastric juice and bile.

The current study presents the case of acute chest and epigastrium pain in a male patient diagnosed with stomach cancer and exhibiting metastases to the lungs, liver and lymph nodes. Written informed consent for the publication of this study was obtained from the patient’s wife.

## Case report

### Scoring systems

Pain was rated by the patient by means of a 0–10 Verbal Rating Scale (VRS) where 0/10 represents no pain and 10/10 represents the worst imaginable pain (21). In addition, a questionnaire concerning symptoms and their intensity was conducted; the patient estimated the intensity of their own pain using a scale of 0–3 (Likert scale), where 0/3 represented no symptoms and 3/3 represented very intense symptoms (21). The patient’s general health status was assessed using the Karnofsky performance status scale (score range, 0–100%) and the Eastern Cooperative Oncology Group (ECOG) scale (score range, 0–5) (21).

### Case presentation

A 41-old male patient diagnosed with stomach cancer, with metastases to the lymph nodes, lungs and liver, who underwent a subsequent GE and postoperative chemotherapy, was admitted to the Palliative Medicine In-patient Unit of the University Hospital of Lord’s Transfiguration (Poznan, Poland). The patient was admitted to the hospice three months following the final chemotherapy course, due to acute chest and epigastrium pain, which had lasted for a number of hours (since morning the same day). The patient complained of a clenching, smothering chest pain (VRS, 10/10), permanent, diffused epigastrium pain (VRS, 10/10) and non-colic left intra-abdominal pain (VRS, 10/10), accompanied by saliva overproduction, nausea (Likert scale, 1/3), regurgitation (Likert scale, 1/3) anorexia (Likert scale, 1/3), fatigue (Likert scale, 1/3), weakness (Likert scale, 1/3), worrying (Likert scale, 2/3), nervousness (Likert scale, 1/3), hopelessness (Likert scale, 2/3), internal tension (2/3) and anxiety (Likert scale, 1/3). In addition, the patient reported a poor quality of life. The patient received the following therapeutic agents prior to admission: Tramadol, 100 mg orally (p.o.) every 4 h; metoclopramide, 10 mg p.o. 3 times/day; diazepam, 5 mg/day p.o.; clorazepate, 10 mg/day p.o.; megestrol, 20 ml/day p.o.; and enoxaparine, 0.4 ml/day (40 mg/day) subcutaneously. The patient refused to undergo the continuous administration of analgesics; instead, the patient only used these agents in the case of severe pain. On the day of admission, prior to arriving at the hospital, the patient self-administered a single dose of morphine sulfate (MF; 20 mg subcutaneously) and immediate-release formulation MF (30 mg p.o.).

The initial examination conducted upon admission revealed that the patient was conscious and coherent, of lean build and capable of walking unaided. The patient was identified to have a Karnofsky score of 50, an ECOG score of three and a pulse oximeter oxygen saturation of 95%. Additional examinations revealed impaired resonance and vesicular murmur at the base of the two lungs, as well as tenderness of the left intra-abdomen and a 7×10-cm diameter intra-abdominal resistance, as determined by palpation. Furthermore, the patient demonstrated signs of peripheral neuropathy. The preceding day’s stool and flatulence were normal, and the patient declared no allergies. Table I presents the patient’s abnormal laboratory test results upon admission.

### Pertinent medical history

The primary site of the cancer was the stomach, which was diagnosed as adenocarcinoma 11 months prior to admission to the University Hospital of Lord’s Transfiguration. Subsequently, cancer esophageal infiltration and metastases to the lungs, liver and lymph nodes were detected. The patient had previously undergone a GE, splenectomy, removal of the greater and lesser omentum, lymphadenotomy followed by chemotherapy and second-line palliative chemotherapy. The final chemotherapy course was conducted three months prior to admission and resulted in the side effect of peripheral polineuropathy. Additionally, one month prior to admission, a control esophagoduodenoscopy revealed no recurrence within anastomosis. The patient, being aware of the diagnosis and prognosis, asked not to be informed of the disease progress by his wife or the medical staff.

### Treatment of the patient upon admission (day 0)

**Figure 1:**
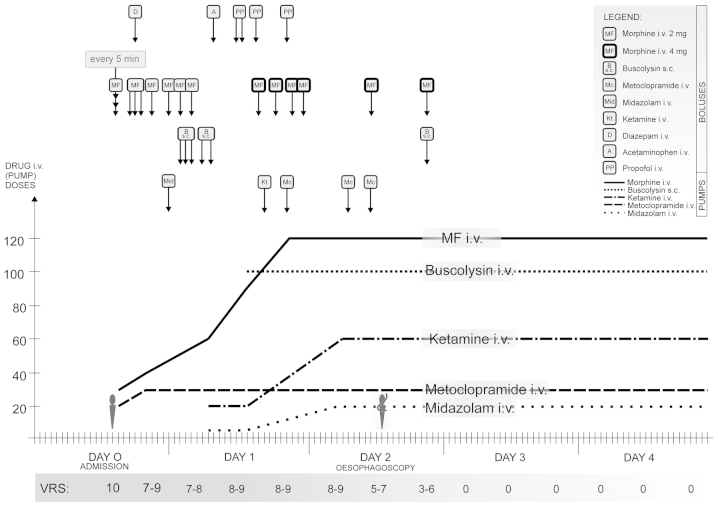
Agents administered to the patient and pain control during the first four days of hospitalization. i.v., intravenous; s.c., subcutaneous; VRS, verbal rating scale.

The patient was administered with fractioned rescue doses of MF [3×2 mg, intravenously, every 5 min] and Spasmalgon^®^ [1 5-ml ampule (amp.) contains 2,500 mg etamizole sodium, 0.1 mg fenpiverine bromide and 10 mg pitofenone hydrochloride; Solpharma, Sofia, Bulgaria]. The administration of 1 amp. Intravenous (i.v.) administration of Spasmalgon caused a decrease in pain of 75% (VRS, 3/10). Subsequently, 30 mg/24 h MF was, continuously infused, intravenously, using a pump (Fig. 1).

### Symptoms, examination and treatment of the patient during hospitalization

On day one, the patient complained of acute chest and epigastrium pain, predominantly paroxysmal, clenching and spasmodic, accompanied by saliva overproduction, dysphagia, nausea and spontaneous or swallowing-induced return of undigested food. The pain was partially refractory to MF administration (Fig. 1). As the symptoms were severe and no malignant recurrence in the anastomosis was observed one month prior to admission, the pain was considered to be of cardiac origin. However, no pathological changes were detected in the electrocardiogram examination and cardiac enzyme levels appeared normal; therefore, acute heart muscle ischemia was excluded as a potential diagnosis.

On day two, esophagoscopy was performed, which considerably improved the pain control (Fig. 1). At midday, the patient was able to walk unaided and use their laptop. The esophagoscopy revealed masses of undigested food moving backwards in the esophagus, which were removed by the examiner. In addition, external pressure was identified on the esophagus. The esophagus, esophagoduodenostomy and jejunum were difficult to assess due to semifluid food remains (despite a previous aspiration of food masses) and small food portions were trapped in the bronchial tree. Following the esophagoscopy, a decompressing tube was installed in the esophagus; due to technical reasons and patient tolerance levels, a decompressing tube of only 5 mm in diameter was installed. Parenteral nutrition (PN; i.v.) and antibiotics [Augmentin (amoxicillin with clavulanic acid), 3×1.2 g/day, i.v.; GlaxoSmithKline, Brentford, UK] were introduced as part of the treatment strategy, due to the development of leukocytosis and interstitial lung changes observed in examinations (Fig. 1). The decompressing tube was not fully successful in removing food and saliva from the esophagus, possibly due to its small diameter. However, the patient’s pain control was sufficient to allow for further examination.

Abnormalities were detected in the echocardiogram examination, including the presence of periaortic masses, which moderately pressed on the left atrium, moving the descending aorta, as was visible in the mediastinum. In addition, abnormalities were detected in the chest computerized tomography (CT) scan, for example sparse, interstitial densities were visible in the basal segments of the two lungs. Abnormalities in the abdominal CT scan included homogenous hepatomegaly (craniocaudal dimension, 20 cm), with numerous sparse, solid, hypotensive focal lesions, which were possibly metastatic lesions. Following splenectomy, enlarged pockets of lymph nodes (24×26 cm; 27×19 cm) were visible in the periaortic space between the celiac trunk and the superior mesenteric artery.

A second esophagoscopy was recommended to clarify the source of the pain and revealed trapped masses of liquid-pulp consistency (of which the majority was removed), no changes in the esophagus, esophagoenterostomy (depth, 43 cm) without pathological changes, possible irregularities in the mucous membrane below the Roux-en-Y anastomosis (detailed assessment impossible due to food remains), 10 cm of intestine with no pathological changes and an irregular mucous membrane at the end of this section. Tissue samples were obtained and used to determine a diagnosis of suspected intestinal cancer recurrence or infiltration to the intestine.

On day three, the patient was able to walk unaided, and personal computer use and pain control were satisfactory (Fig. 1). On day four, the decompressing tube was removed on the patient’s demand, due to the complaint of a sore throat. However, chest and epigastrium pain were satisfactorily controlled (Fig. 1) and intravenous feeding was introduced as an alternative. The patient was discharged from the hospital at his request, and received home hospice care. The patient’s condition gradually deteriorated and the patient succumbed to the disease five days following the in-patient unit stay.

## Discussion

In the present case, the disease dynamics, progression and symptoms were acute. Therefore, investigating the cause of the pain was crucial for satisfactory symptom control, particularly for pain management. Esophageal diseases and/or dysfunctions are the most common causes of angina-like chest pain (22), and the development of esophageal and cardiac pains may overlap (23) and, thus, cardiac and esophageal chest pains frequently cannot be differentiated in the case history. Among cancer patients, ≤50% experience pain that they perceive as moderate or severe, while 30% experience severe pain. Furthermore, 25% of cancer patients approach mortality in pain (24). The present case demonstrates that diagnostic procedures are essential in the end-of-life period. A radical improvement was observed subsequent to esophagoscopy, due to the removal of the food masses from the esophagus. As the patient examination conducted one month prior to hospital admission revealed no recurrence of malignancy, the acute chest and epigastrium pain was considered to be of cardiac origin. However, once this source of pain was excluded, the metastases were considered to have caused the pain. Finally, esophagitis and esophageal wall distention were identified to be the true source of the patient’s angina-like, spasmodic pain.

We aimed to respond to the patient’s needs, while controlling the condition and anticipating possible drug-drug interactions; thus, the drug doses had to be meticulously adjusted and titrated. Calcium channel blockers and nitrates were excluded from the treatment strategy of the present patient due to the patient experiencing problems swallowing calcium channel blocker pills and having a blood pressure too low for nitroglycerin administration (i.v. or in aerosol), respectively, as well as unwanted effects (dizziness due to hypertension, headache and flushing). The treatment required a balance between controlling the symptoms and maintaining therapeutic safety. The patient was provided with an optimal quality of life.

In the conclusion, the present study determined that a cancer patient in palliative care, approaching mortality, may require a secondary diagnosis to provide an appropriate quality of life and preserve the patient’s dignity. Additionally, pain treatment should be adjusted according to anatomical, pathophysiological and pharmacological factors. In the present case, the treatment of acute epigastric and chest pain originating from cancer was posed risks due to the parenteral co-administration of multiple strong-acting agents, however, the therapy was unavoidable. Careful drug titration and treatment monitoring is, therefore, essential for the safe administration of multidrug pain control. It was also concluded that a patient exhibiting regurgitation of food and experiencing severe pain requires constant monitoring and nursing care, for example due to the risk of developing aspiration pneumonia. For the present patient, esophagoduodenoscopy proved to be both diagnostic and therapeuticas it allowed for emptying the esophagus and satisfactory pain control. The second esophagoduodenoscopy was also diagnostic as it revealed changes accounting for intestinal cancer recurrence or infiltration to intestine.

## Figures and Tables

**Table I:** Abnormalities in laboratory analyses in the patient upon presentation.

Variable	Value (reference value)
White blood cells, 10E9/l	12.81 (4.00–10.00)
Hemoglobin, mM	7.50 (7.45–10.00)
Hematocrit l/l	0.37 (0.36–0.47)
Platelets, 10E9/l	465.00 (130–390)
Serum glucose, mM	13.21 (3.90–5.60)
Serum magnesium, mM	0.70 (0.74–0.99)
γ-glutamyl transpeptidase, U/l	124.00 (<55)
Lactate dehydrogenase, U/l	600.00 (105–330)
